# Enhanced Sensitivity of Binary/Ternary Locally Resonant Porous Phononic Crystal Sensors for Sulfuric Acid Detection: A New Class of Fluidic-Based Biosensors

**DOI:** 10.3390/bios13070683

**Published:** 2023-06-27

**Authors:** Khaled Aliqab, Hussein A. Elsayed, Meshari Alsharari, Ammar Armghan, Ashour M. Ahmed, Ahmed Mehaney

**Affiliations:** 1 Department of Electrical Engineering, College of Engineering, Jouf University, Sakaka 72388, Saudi Arabia; kmaliqab@ju.edu.sa; 2 Physics Department, Faculty of Science, Beni-Suef University, Beni-Suef 62512, Egypt; hussien.abdelghani@science.bsu.edu.eg (H.A.E.); ashour.mohamed@science.bsu.edu.eg (A.M.A.); ahmed011236@science.bsu.edu.eg (A.M.); 3 Physics Department, College of Science, Imam Mohammad Ibn Saud Islamic University (IMSIU), Riyadh 11623, Saudi Arabia

**Keywords:** porous silicon, resonant modes, phononic crystals, sulfuric acid, liquid sensor, acoustic wave, temperature

## Abstract

This research presented a comprehensive study of a one-dimensional (1D) porous silicon phononic crystal design as a novel fluidic sensor. The proposed sensor is designed to detect sulfuric acid (H_2_SO_4_) within a narrow concentration range of 0–15%. Sulfuric acid is a mineral acid extensively utilized in various physical, chemical, and industrial applications. Undoubtedly, its concentration, particularly at lower levels, plays a pivotal role in these applications. Hence, there is an urgent demand for a highly accurate and sensitive tool to monitor even the slightest changes in its concentration, which is crucial for researchers. Herein, we presented a novel study on the optimization of the phononic crystal (PnC) sensor. The optimization process involves a comparative strategy between binary and ternary PnCs, utilizing a multilayer stack comprising 1D porous silicon (PSi) layers. Additionally, a second comparison is conducted between conventional Bragg and local resonant PnCs to demonstrate the design with the highest sensitivity. Moreover, we determine the optimum values for the materials’ thickness and number of periods. The results revealed that the ternary local resonant PnC design with the configuration of {silicone rubber/[PSi1/PSi2/PSi3]^N^/silicone rubber} is the optimal sensor design. The sensor provided a super sensitivity of 2.30 × 10^7^ Hz for a concentration change of just 2%. This exceptional sensitivity is attributed to the presence of local resonant modes within the band gap of PnCs. The temperature effects on the local resonant modes and sensor performance have also been considered. Furthermore, additional sensor performance parameters such as quality factor, figure of merit, detection limit, and damping rate have been calculated to demonstrate the effectiveness of the proposed liquid sensor. The transfer matrix method was utilized to compute the transmission spectra of the PnC, and Hashin’s expression was employed to manipulate the porous silicon media filled with sulfuric acid at various concentrations. Lastly, the proposed sensor can serve as an efficient tool for detecting acidic rain, contaminating freshwater, and assessing food and liquid quality, as well as monitoring other pharmaceutical products.

## 1. Introduction

Sulfuric acid (H_2_SO_4_), an odorless and colorless aqueous solution, has garnered significant attention in the industrial chemistry community [1,2]. It has a density of 1.83 g/cm^3^ and a molar mass of 98.1 g/mol [1]. Over the past decades, H_2_SO_4_ has been widely utilized in various industries and applications, including batteries, fertilizers, resin manufacturing, metal processing, pharmaceutical products, mineral dissolution, cleaning products, and catalytic processes [2]. However, H_2_SO_4_ is a primary contributor to environmental problems such as acidic rain [2,3,4]. Hence, there is an urgent need for an efficient, accurate, and simple method to detect and monitor H_2_SO_4_.

In recent decades, numerous methods and techniques have emerged for this purpose [2,3,4,5,6,7,8,9,10]. Absorption spectroscopy techniques, known for their high sensitivity, fast response, accuracy, and non-contact measurement, have been extensively employed for detecting liquids and gases at different concentrations [4]. The detection and monitoring of H_2_SO_4_ have shown significant advancements through mid-infrared spectroscopy, exploiting its abundant vibrational absorption in this spectral range [5,6,7]. Zhou et al. also explored UV absorption spectroscopy to detect H_2_SO_4_ solutions within the wavelength range of 185 nm to 210 nm [4]. They observed a prominent absorption peak at 193 nm, with its intensity increasing significantly as the H_2_SO_4_ concentration rises.

Moreover, Maidi et al. suggested a novel design based on the photonic crystal fiber (PCF) for detecting aqueous H2SO4 of different concentrations [3]. However, the techniques and methods mentioned above may encounter with some drawbacks in the vicinity of the high fabrication cost, limited accuracy, fragility, fabrication complexity, and limited use [3,10]. Therefore, resorting to new techniques, methods, or structures could be of potential interest towards simple, accurate, and low-cost detection.

In this regard, new classes of multilayered nano and microstructures, including photonic and phononic crystals, are recently considered as chemical, physical, and biomedical sensors. Photonic crystals (PCs), characterized by their periodic modulation, can manipulate incident electromagnetic waves [11,12,13,14,15]. These designs have demonstrated relatively high performance in detecting various phenomena such as Fano resonance (FR) [16], resonant defect modes [17], Tamm plasmon resonance (TPR) [18,19], and surface plasmon resonance (SPR), as well [20,21,22]. In contrast, phononic crystals (PnCs) are introduced as periodic artificial microstructures to confine the propagation of acoustic and elastic waves [23,24]. Due to their periodicity and mismatched acoustic properties (mass densities and elastic constants of PnC materials), PnCs have the potential to create stop frequency bands that completely control the acoustic wave response [24,25]. These bands are now referred to as phononic bandgaps (PnBGs) [23,24,25,26,27,28]. Interestingly, PnBGs can manifest in one, two, or three dimensions depending on the periodicity of the PnC structure [26,27]. Moreover, the localization of phonons with specific frequencies can be achieved through the PnBG of disordered PnC designs [29]. Furthermore, PnCs show promise in the design and fabrication of various industrial and technological devices, including acoustic signal processing [30], filters [31,32], elastic topological insulators [33], demultiplexers [34], and modulators [35]. Moreover, various PnC structures have been widely introduced in sensing and detection applications [36,37,38,39,40,41]. In this regard, Fang et al. have developed a high-quality and ultra-sensitive sensing tool based on a 2D PnC structure to distinguish different concentrations of acetone [36]. Furthermore, Mehaney et al. have discussed the role of 2D PnCs as demultiplexers for detecting three different heavy metals (CuSO_4_, MgSO_4_, and MnSO_4_) simultaneously [37]. The suggested design can detect extremely low concentrations of these pollutants in freshwater. Additionally, Rostami-Dogolsara et al. have proposed an acoustic demultiplexer based on fork-shaped PnCs for determining the compositions of liquid mixtures [38]. Moreover, Lucklum has experimentally demonstrated the detection of sodium chloride and glucose concentrations in aqueous solutions using solid-air 3D PnCs that confine a fluidic cavity resonator [39]. Furthermore, PnC structures based on the 1D configuration have garnered considerable interest in the detection and sensing of various liquids at both theoretical and experimental levels [40,41]. However, a significant challenge during the fabrication of the suggested designs in 1D, 2D, and 3D may arise from the inclusion of a cavity or defect layer. Specifically, the fabrication tolerance of the cavity or defect layer thickness can significantly impact the sensor’s performance. Moreover, fabricating 2D and 3D PnC structures may pose additional difficulties compared to 1D designs. In this communication, our objective is to present a simple, efficient, and high-performance sensor for monitoring and detecting aqueous solutions of H_2_SO_4_ within a narrow concentration range. Our approach is based on the design of a one-dimensional (1D) ternary PnC made of porous silicon, which includes two silicon rubber buffering layers. Each unit cell of the designed structure comprises three porous silicon layers of different porosities and thicknesses. The choice of ternary configuration in each unit cell of the PnC structure is due to its role in improving the performance of the candidate sensor compared to the binary one [42,43]. Moreover, the choice of silicon rather than epoxy and nylon is due to its excellent moisture resistance, high hardness, chemical stability, and low coefficient of thermal expansion, as well. These properties are up-and-coming to meet the application needs.

Interestingly, the presence of two silicon rubber layers gives rise to certain local resonant (LR) modes within the PnBG. Here, the detection method is essentially based on the position of the LR mode. Notably, the position of this mode is significantly affected, as the pores are filled with the aqueous solution of H_2_SO_4_. Furthermore, the LR mode could be of interest regarding a relatively high performance in the vicinity of high sensitivity and low detection limit compared to the conventional resonant defect modes [44]. In addition, tolerance errors during the cavity or defect layer thickness fabrication may significantly influence the candidate sensor’s performance [41]. Therefore, the dependence on the emergence of LR mode could be crucial towards detecting and monitoring H_2_SO_4_ despite the most negligible fluctuations in its concentration. On the other hand, the use of porous silicon in the design of our suggested sensing tool could be of significant interest due to its numerous advantages. Firstly, porous silicon serves as an excellent alternative for the cavity or defect layer, enabling easy and smooth investigation of the detection process. Secondly, the fabrication of 1D porous silicon PnCs with micrometer-scale thicknesses can be readily achieved [45,46]. Additionally, incorporating porous silicon in the fabrication of PnCs may offer a viable solution to the problem of mismatching between different layers or materials in the designed structure. Furthermore, the utilization of porous silicon in PnC designs presents certain advantages compared to periodic structures and photonic crystals, particularly concerning fabrication tolerance. Notably, the fabrication of porous silicon photonic crystals at the nanometer scale requires highly sensitive techniques, unlike PnC structures. Lastly, porous silicon with a porosity of nearly 80% or less still exhibits acceptable mechanical properties [46]. Thus, we believe that the introduction of porous silicon in the design of 1D PnCs for detecting H_2_SO_4_ concentration could provide several advantages over other conventional approaches. Meanwhile, the numerical findings simulate the impact of H_2_SO_4_ concentration and temperature on the performance of the sensor under consideration. In the following section, we present the design of a 1D ternary PnC sensor along with its relevant parameters. Additionally, we provide a theoretical framework that explains the response of the acoustic waves, demonstrated by plotting the transmission spectra against each H_2_SO_4_ concentration.

## 2. Sensor Design and Theoretical Background

### 2.1. The Candidate Sensing Tool

**Figure 1:**
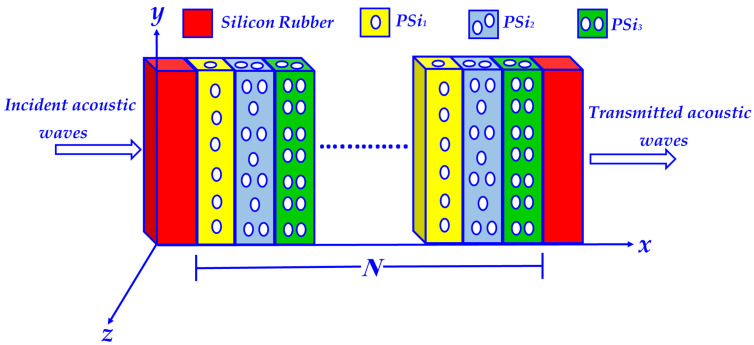
A three-dimensional schematic diagram of the candidate 1D ternary PnC structure immersed between two identical layers of silicon rubber.

In this subsection, we have presented the key features of the designed sensing tool. The 1D ternary PnC structure is placed between two identical layers of silicon rubber, which are responsible for the emergence of the LR mode. Each unit cell of the designed 1D ternary PnC sensor comprises three layers of porous silicon with varying characteristics of porosity (PSi_1_, PSi_2_, and PSi_3_) and thicknesses (d_1_, d_2_, and d_3_). In this regard, an aqueous solution of H_2_SO_4_ of different concentrations can flow through these pores. Therefore, our candidate sensor can be configured as, {silicon rubber/[PSi_1_/PSi_2_/PSi_3_]^N^/silicon rubber} as shown in Figure 1. Additionally, Table 1 summarizes the mechanical properties of materials in terms of densities, acoustic sound speed, and thicknesses [45,46,47].

### 2.2. Theoretical Methodology

This subsection presents a comprehensive visualization of the interacting acoustic waves within the candidate sensing device. The demonstration of this interaction is particularly evident in the context of transmittance and reflectance properties. Over the past few decades, numerous approaches and methods have emerged to describe this interaction, such as the transfer matrix approach [48,49,50,51] and the finite element method [36,37,38,39]. Nonetheless, the 2 × 2 transfer matrix approach is considered the most straightforward and accurate for the 1D photonic and phononic crystal (PnC) designs. Therefore, we introduce a detailed visualization of this method through the upcoming steps.

Meanwhile, Figure 1 illustrates that the upcoming acoustic waves propagate perpendicularly on the prospective 1D ternary PnC structure, where the constituent layers are stacked along the x-direction. As the propagating acoustic waves interact with the desired design, they can be dispersed through multiple waves. Therefore, the density and wave speed through the structure layers can be affected. In this context, the response of the propagating acoustic waves through the desired design could be described using the following Equation [49,50,51]:

(1) $$\frac{1}{C_{j}^{2}}\frac{\partial^{2}p}{\partial t^{2}}-\nabla^{2}p=0$$

where C_i_ defines the longitudinal sound speed within layer j, and p describes the pressure of the acoustic wave. Then, the solution of Equation (1) can be simply introduced in the following manner [44]:

(2) $$p_{j}=\left(A_{+}^{\left(j\right)}e^{+iK_{j}X}+A_{-}^{\left(j\right)}e^{-iK_{j}X}\right)e^{i\omega t}$$

Here, $A_{+}^{\left(j\right)}$ and $A_{-}^{\left(j\right)}$ are the amplitudes of the transmitted and reflected waves, respectively, ω describes the frequency of the propagating acoustic waves, and $K_{j}=\omega /C_{j}$ defines the wave vector, which could be varied due to the changes in the acoustic sound speed across the structure layers. Then, the applied stress and wave displacement continuity are expected at the border between every two adjacent layers. Therefore, the stress introduced due to the propagating acoustic waves through the desired design can be defined as [52,53,54]:

(3) $$\sigma =E_{j}\frac{\partial p_{j}}{\partial x}$$

Such that, $E_{j}$ Refers to Young’s modulus of a specified layer j. Thus, by combining Equations (2) and (3), the stress can be introduced as [44,52]:

(4) $$\begin{array}{rr} & \sigma \left(x\right)=iZ_{j}\left[A_{+}^{\left(j\right)}e^{+iK_{j}X}-A_{-}^{\left(j\right)}e^{-iK_{j}X}\right] \end{array}$$

where $Z_{j}=E_{j}K_{j}$ describes the acoustic impedance. Then, wave displacement and stress can be described in a generalized matrix form [44,49]:

(5) $$\left[\begin{array}{l} u(x)\\ \sigma (x) \end{array}\right]=\left[\begin{array}{cc} 1 & 1\\ iZ_{j} & iZ_{j} \end{array}\right]\left[\begin{array}{c} A_{+}^{\left(j\right)}e^{+iK_{j}X}\\ A_{-}^{\left(j\right)}e^{-iK_{j}X} \end{array}\right]=W_{j}\left[\begin{array}{c} A_{+}^{\left(j\right)}e^{+iK_{j}X}\\ A_{-}^{\left(j\right)}e^{-iK_{j}X} \end{array}\right]$$

Here, $W_{j}$ indicates the wave matrix at the border between two adjacent layers. For $X_{R}^{j}=X_{L}^{j}+d_{j}$ such that $X_{R}^{j}$ and $X_{L}^{j}$ clarify the left and right border of each layer (j), respectively; therefore, we have [53]:

(6a) $$\begin{array}{rr} & \left[\begin{array}{c} u\left(X_{R}^{j}\right)\\ \sigma \left(X_{R}^{j}\right) \end{array}\right]=\left[\begin{array}{cc} e^{+iK_{j}d_{j}} & 0\\ 0 & e^{-iK_{j}d_{j}} \end{array}\right]W_{j}\left[\begin{array}{c} A_{+}^{\left(j\right)}e^{+iK_{j}}x_{L}^{j}\\ A_{-}^{\left(j\right)}e^{-iK_{j}X_{L}^{j}} \end{array}\right]=Q_{j}W_{j}\left[\begin{array}{c} A_{+}^{\left(j\right)}e^{+iK_{j}x_{L}^{j}}\\ A_{-}^{\left(j\right)}e^{-iK_{j}X_{L}^{j}} \end{array}\right] \end{array}$$

(6b) $$\left[\begin{array}{c} u\left(X_{L}^{j}\right)\\ \sigma \left(X_{L}^{j}\right) \end{array}\right]=W_{j}\left[\begin{array}{c} A_{+}^{\left(j\right)}e^{+iK_{j}x_{L}^{j}}\\ A_{-}^{\left(j\right)}e^{-iK_{j}x_{L}^{j}} \end{array}\right]$$

Here, Q_j_ describes the propagating matrix through a distinct layer j of thickness $d_{j}$. Then, Equations (6a) and (6b) can be combined such that [52]:

(7) $$\left[\begin{array}{l} u\left(X_{R}^{j}\right)\\ \sigma \left(X_{R}^{j}\right) \end{array}\right]=Q_{j}W_{j}\left[\begin{array}{c} A_{+}^{\left(j\right)}e^{iK_{j}X_{L}^{j}}\\ A_{-}^{\left(j\right)}e^{-iK_{j}X_{L}^{j}} \end{array}\right]=W_{j}Q_{j}W_{j}^{-1}\left[\begin{array}{c} u\left(X_{L}^{j}\right)\\ \sigma \left(X_{L}^{j}\right) \end{array}\right]=m_{j}\left[\begin{array}{c} u\left(X_{L}^{j}\right)\\ \sigma \left(X_{L}^{j}\right) \end{array}\right]$$

where governs the transfer matrix through a given layer j, such that [52]:

(8) $$m_{j}=\left[\begin{array}{cc} cos\left(K_{j}d_{j}\right) & 1/Z_{j}sin\left(K_{j}d_{j}\right)\\ -Z_{j}sin\left(K_{j}d_{j}\right) & cos\left(K_{j}d_{j}\right) \end{array}\right]$$

Thus, Equation (8) can be generalized over all the layers of the designed structure. Then, the final matrix describing the response of the acoustic waves inside the whole network that contains n layers is given as [52]:

(9) $$M=\left(\begin{array}{cc} M_{11} & M_{12}\\ M_{21} & M_{22} \end{array}\right)=\prod_{i=1}^{n}W_{j}Q_{j}W_{j}^{-1}=\prod_{i=1}^{n}m_{j}$$

Therefore, the transmission coefficient of the desired 1D PnC structure can be computed in the vicinity of the elements of matrix M as [44]:

(10) $$\frac{U_{s}}{U_{0}}=\frac{2E_{0}\left(M_{11}M_{22}-M_{12}M_{21}\right)}{E_{0}\left(M_{11}-E_{s}M_{21}\right)-\left(M_{12}-E_{s}M_{22}\right)}$$

Such that, $U_{0},U_{s}$ refer to the incident and transmitted waves amplitudes, respectively, and $E_{0}$ and $E_{s}$ describe Young’s moduli at the left and right borders of the designed PnC structure.

### 2.3. Porous Silicon Acoustic Velocity and Density

In this subsection, we have introduced the equations that govern the acoustic velocity and density of PSi. In particular, the change in the analyte’s type and concentration is also significantly effective on the acoustic properties of PSi. Meanwhile, the rule of the mixture can be utilized to describe the average mass density of each PSi as the analyte flows through it such that [45,46]:

(11) $$\rho_{j}^{\prime }=\rho_{l}R_{j}+\rho_{0}(1-R_{j})$$

Here, $\rho_{0}$ (= 2.33 g/cm^3^) describes the density of the non-porous silicon, $\rho_{l}$ defines the liquid density, and $R_{j}$ indicates the porosity of a given layer j. Then, as the pores are filled with the proposed analyte rather than voids, the effective acoustic velocity of a given PSi layer j is written as [45]:

(12) $$c_{lj}^{\prime }=\sqrt{\frac{\lambda_{j}^{\prime }+{2\mu }_{j}^{\prime }}{\rho_{j}^{\prime }}}$$

where, $\lambda_{j}^{\prime }$ and $\mu_{j}^{\prime }$ yield for the effective Lame’s constants of a specified layer j. These parameters could be described in terms of porosity based on Hashin and Shtrikman theory [45] as follow:

(13a) $$\frac{{\left(1-R_{j}\right)}^{2}}{\left(1+b_{\lambda }R_{j}\right)}\lambda_{0}$$

(13b) $$\mu_{j}^{\prime }=\frac{{\left(1-R_{j}\right)}^{2}}{\left(1+b_{\mu }R_{j}\right)}\mu_{0}$$

Such that, $\lambda_{0}$ and $\mu_{0}$ are the Lame’s constants without porosity, i.e., R_j_ = 0 and $b_{\lambda }$ and $b_{\mu }$ are constants related to Poisson’s ratio of bulk silicon ($\nu_{0}$= 0.265), where [45]:

(14a) $$b_{\lambda }=\frac{\left(1+\nu_{0}\right)}{2\left(1-2\nu_{0}\right)}$$

(14b) $$b_{\mu }=\frac{\left(11-19\nu_{0}\right)}{4(1+\nu_{0})}$$

### 2.4. Acoustic Properties of H_2_SO_4_ Aqueous Solution

**Figure 2:**
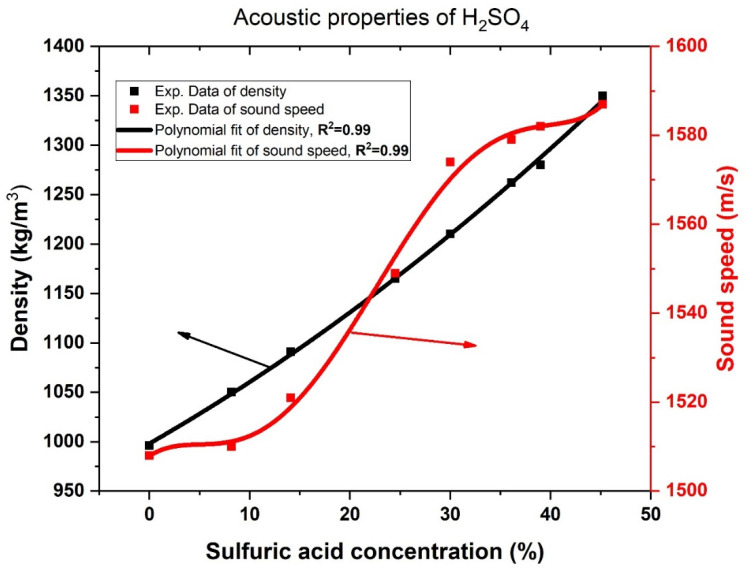
Acoustic properties (density and sound speed) of H_2_SO_4_ aqueous solution versus its concentration at 30 °C.

Finally, we present in this subsection the acoustic properties of the H_2_SO_4_ aqueous solution based on the variations in its concentration. Meanwhile, Figure 2 displays the response of both sound speed and density of H_2_SO_4_ versus its concentration at 30 °C. This response is obtained based on some numerical experimental measurements fitted to describe this variation [47]. Therefore, the acoustic speed and density of the H_2_SO_4_ aqueous solution can be described as:

(15) $$\rho (\frac{kg}{m3})=998.132+5.81056\;C+0.04126\;C^{2}$$

(16) $$v\left(\frac{m}{s}\right)=1507.85313+1.53646\;C-0.33427\;C^{2}+0.03031\;C^{3}-0.000844\;C^{4}$$

In this regard, Figure 2 clarifies that the density of the aqueous solution increases linearly with the increase in the concentration of H_2_SO_4_. Actually, the data reported in Figure 2 was reproduced based on the experimental verification that was demonstrated in reference [47]. Meanwhile, the density increases from 998.132 at 0 concentration to 1343.1587 at a concentration of 45%. H_2_SO_4_ salt is specified with a relatively high density (1830 Kg/m^3^) compared to pure water. Thus, the flow of H_2_SO_4_ salt through pure water significantly increments in the mass per unit volume of the aqueous solution due to the changes in the particle composition through a given volume.

In contrast, the acoustic wave velocity of the aqueous solution takes a nonlinear response regarding the increase of H_2_SO_4_ concentration. Such response can be numerically described based on a polynomial fitted equation of the 4th degree as depicted in Equation (16). As listed in Table 1, the acoustic speed of H_2_SO_4_ salt is equivalent to 1253 m/s, which is smaller than that of pure water (1507 m/s). Therefore, adding H_2_SO_4_ salt is significantly effective on the acoustic wave velocity of the aqueous solution. Therefore, the dramatic response of the density and acoustic speed of the H_2_SO_4_ aqueous solution could be of potential interest during the detection procedure of H_2_SO_4,_ whatever its concentration, as discussed in the upcoming section.

## 3. Numerical Verifications and Discussions

### 3.1. Transmission Spectra of the Proposed Sensor Design

This section investigates the effect of H_2_SO_4_ solution at various concentrations on the transmission spectra of 1D-PSi-PnCs. The H_2_SO_4_ solution can flow through the pores of PSi layers. For comparison, binary and ternary 1D-PSi-PnCs multilayers are presented and discussed. The PSi multilayers are sandwiched between two identical rubber layers to generate local resonant (LR) modes through the PnBG. Additionally, the conventional Bragg 1D-PSi-PnCs multilayer without rubber layers, which does not support resonance modes, is introduced and studied. For numerical purposes, the frequency used in all calculations was normalized to the acoustic sound speed in the PSi_2_ layer (C = 7.0883 × 10^3^ m/s) when filled with pure water (0% concentration). The normalized frequency can be expressed as ωa/2πC. The transmittance spectrum is plotted as a function of the normalized frequency.

### 3.2. Binary Structure

**Figure 3:**
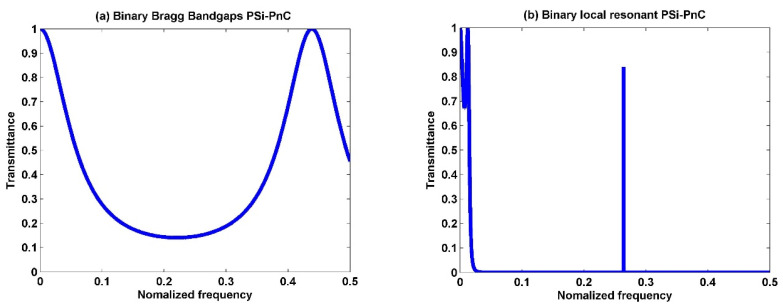

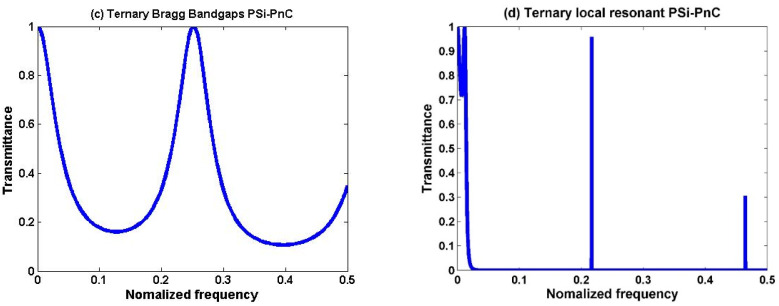
The transmission spectra of (**a**) Binary Bragg Bandgaps PSi-PnC [PSi_1_/PSi_2_]^2^, (**b**) Binary local resonant PSi-PnC [Silicone rubber/[PSi_1_/PSi_2_]^2^/Silicone rubber], (**c**) Ternary Bragg Bandgaps PSi-PnC [PSi_1_/PSi_2_/PSi_3_]^2^, and (**d**) Ternary local resonant PSi-PnC [Silicone rubber/[PSi_1_/PSi_2_/PSi_3_]^2^/Silicone rubber]. All figures are at a concentration of 0% of H_2_SO_4_.

Assume binary 1D-PSi-PnCs containing two-unit cells; each unit cell comprises two different PSi layers. The first material is PSi_1_ (P = 20%), and the second is PSi_2_ (P = 40%). Figure 3a shows the transmission spectra of the binary Bragg bandgap, which consists of [PSi_1_/PSi_2_]^2^. Figure 3b shows the transmission spectra of the binary local resonant 1D-PSi-PnC, which consists of Silicone rubber/[PSi_1_/PSi_2_]^2^/Silicone rubber. It is important to note that the pores of the PSi layers were filled with pure water (0% concentration). The transmission spectrum of [PSi_1_/PSi_2_]^2^, shown in Figure 3a, exhibits a broad region with low transmission intensity, measuring less than 0.2. Consequently, wave transmission through the PnC is significantly limited within this region. The elastic properties of the PSi_1_ and PSi_2_ layers are close to each other. This means there is no significant acoustic mismatch between the two layers, resulting in no apparent band gaps appearing in the spectrum [55]. Additionally, no transmission resonant modes are observed throughout the [PSi_1_/PSi_2_]^2^ structure’s spectrum in the considered frequency ranges.

Figure 3b demonstrates an intriguing phenomenon in the transmission spectrum of the [PSi_1_/PSi_2_]^2^ structure when two rubber layers are attached to its top and bottom sides. The resulting Silicone rubber/[PSi_1_/PSi_2_]^2^/Silicone rubber structure exhibits two complete PnBGs across the entire range of normalized frequencies. Within the band gap, the transmissivity of the incident acoustic waves is zero, resulting in a stopband where any acoustic waves cannot propagate through the structure and are effectively attenuated [44]. A transmission mode is also observed with narrow broadening and high sharpness. This resonant mode is located at the normalized frequency of 0.216400514 (about 1.70 × 10^8^ Hz), with an intensity of 0.85.

As it is well-known, two mechanisms cause the formation of band gaps: Bragg scattering and local resonance. Bragg scattering occurs when the periodicity of the crystal’s structure causes the incoming wave to interfere constructively or destructively with the scattered wave. This can lead to destructive interference between waves travelling in opposite directions, resulting in a band gap [56]. The width and position of the band gap depend on the crystal’s geometry, the constituent materials’ elastic properties, and the angle of incidence. The associated wavelength with the phononic band gap needs to be of the same order as the periodicity of the structure. This means a huge lattice constant is required to obtain phononic band gaps for a low-frequency range, which limits applications.

Local resonance occurs when a resonant structure is created within the crystal due to the combination of high and low elastic constants in its constituent materials [57,58]. The associated wavelength needs to be two orders of magnitude smaller than the Bragg band gap to achieve local resonance. A locally resonant band gap is related to the resonance frequency associated with the scattering units and depends less on the periodicity and symmetry of the structure. This means that it is possible to overcome the limitation of Bragg band gaps and allow band gaps at low frequencies.

The Bragg diffraction law cannot explain the intense wave localization mode observed in Figure 3b, but rather by the local resonance mechanism. The local resonance mode is generated inside the PnCs due to the high elastic constant of the PSi layers in combination with the low elastic constants of the coating rubber. This generates a strong resonant mode that attenuates waves within the bandgap, resulting in a high degree of wave attenuation. The localized resonance modes create localized regions of intense acoustic energy. These modes effectively trap and guide the wave through the crystal, allowing for precise control propagation of acoustic waves. The local resonant modes of phononic crystals have been extensively studied in recent years due to their unique properties and potential applications [59,60]. These applications include acoustic insulation, noise reduction, vibration damping, ultrasonic transducers, acoustic filtering, waveguiding, acoustic sensing, and medical imaging [61,62].

### 3.3. Ternary Structure

The ternary 1D-PSi-PnC can be obtained by immersing the third layer (PSi_3_ with P = 60%) after the two layers used in binary ones. A very wide peak appeared in the middle of the spectrum at the normalized frequency of approximately 0.25 in ternary 1D-PSi-PnC without adding rubber layers, as seen in Figure 3c. The wide peak mode observed in the ternary structure is due to two interfaces between the three materials, as opposed to the single interface in the binary crystal. Hence, an additional interface in the ternary structure leads to more interference of waves at each interface, resulting in the broader peak mode observed in the transmission spectrum.

In the field of sensors, a broader peak in the transmission spectrum is generally considered undesirable. This is because it can make it more difficult to discern changes in the frequency of the detected acoustic wave. It can result in a lower signal-to-noise ratio and reduced sensitivity to changes in external stimuli. Additionally, a broader peak in the transmission spectrum typically indicates lower localized energy within the crystal. This is problematic for sensing applications that require high sensitivity to small environmental changes. In such cases, the lower energy within the crystal may not be sufficient to generate a detectable response to the external stimulus.

Figure 3d displays the transmission spectrum of a ternary [PSi_1_/PSi_2_/PSi_3_]^2^ PnC with two rubber layers bonded to each side. The spectrum reveals the presence of two distinct and well-defined acoustic modes. The first peak, located at 0.22, exhibits an intensity of 0.98, while the second peak, positioned at 0.46, demonstrates an intensity of 0.29. It is worth noting that the binary structure has one mode, while the ternary structure has two modes in the same frequency range. This is attributed to two interfaces between the three materials in the ternary structure, resulting in more complex wave behavior. This significant discovery implies that the ternary structure offers a wider range of acoustic modes compared to the binary structure. Consequently, this finding holds implications for the design and optimization of phononic crystals across various applications.

### 3.4. Effect of H_2_SO_4_ Concentration

**Figure 4:**
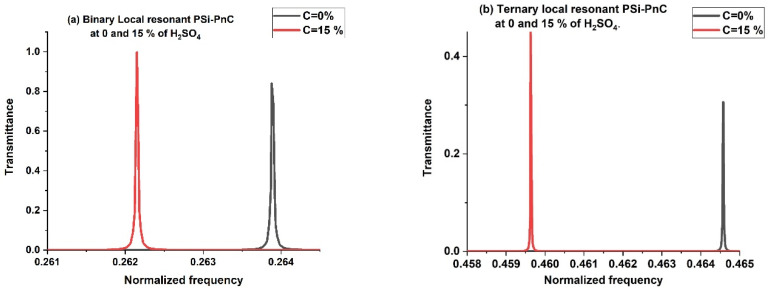
The transmission spectra of (**a**) Binary local resonant PSi-PnC at 0 and 15% of H_2_SO_4_, and (**b**) Ternary local resonant PSi-PnC (right peak) at 0 and 15% of H_2_SO_4_.

The presence of liquid inside the pores of PSi layers can significantly affect wave transmission in PnCs. This subsection presents a comparative study of the sensitivity of a proposed sensor using binary and ternary PnCs with local resonance. Figure 4a,b shows the transmission spectra of two PnCs with identical geometrical structures but different numbers of layers (binary and ternary) at various concentrations of H_2_SO_4_. The proposed structures consist of rubber/[PSi_1_/PSi_2_]^2^/rubber and rubber/[PSi_1_/PSi_2_/PSi_3_]^2^/rubber. The concentration of H_2_SO_4_ is varied from 0 to 15%. The H_2_SO_4_ concentration (C %) and the position peak (f, Hz) are the input signals of the sensor and the output value, respectively. Hence, the sensitivity can be defined by the following equation,

(17) S=∆f/∆C (Hz/%)

In the binary structure, the position of the resonant mode is observed to shift from 0.2638 to 0.262 as the concentration of H_2_SO_4_ varies from 0 to 15%. This shift towards lower frequency is attributed to the increase in the acoustic properties of PSi layers with increasing H_2_SO_4_ concentration, as shown in Figure 2. Additionally, the intensity of the resonant mode is observed to increase, as shown in Figure 4a. A high amplitude of the wave results in a low damping coefficient and strong acoustic wave confinement. The sensitivity of the binary structure to detect changes in H_2_SO_4_ concentration is S = 9.0667 × 10^6^ Hz.

Figure 4b shows that the ternary local resonant PSi-PnC (right peak) exhibited a transfer from 0.42638 to 0.4262 with varying H_2_SO_4_ concentrations, resulting in a sensitivity of S = 1.9667 × 10^7^ Hz. In comparison, the sensing performance of the binary PnC system was lower than the ternary structure. This is because the acoustic parameter changes for propagating waves within binary structures are typically very small and can be ignored. In contrast, the ternary structure offers a richer spectrum of modes, improving sensing capabilities.

From Figure 4, it is evident that the transmission peak of the binary structure is higher than that of the ternary structure. This difference can be attributed to the fact that the two structures have different numbers of layers. Specifically, the ternary structure has more layers than the binary structure, which means it has more interfaces between the layers. This may result in an increase in the amount of reflection, leading to a reduction in the amount of wave that passes through the structure. As a result, the ternary structure exhibits a lower transmission peak.

### 3.5. Performance Study

**Figure 5:**
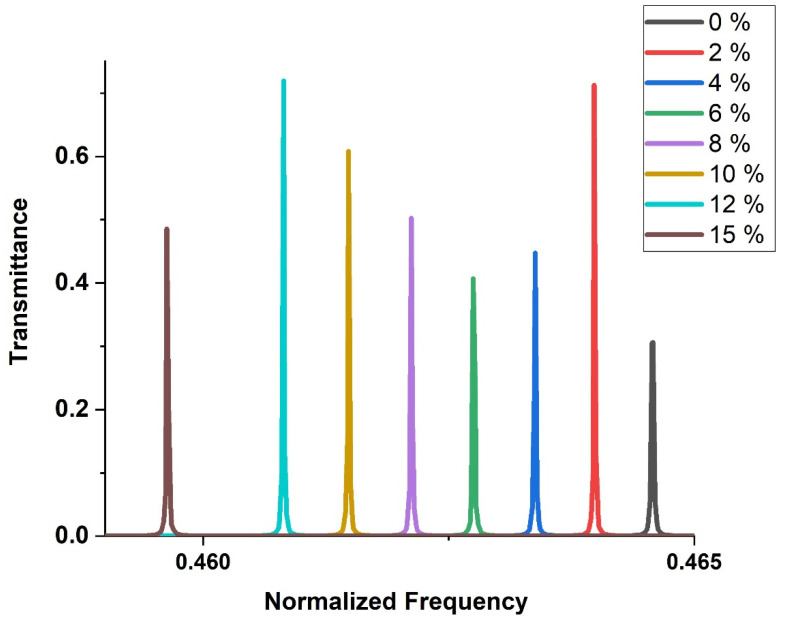
The transmission spectra of the ternary local resonant PSi-PnC at different concentrations of H_2_SO_4_.

The transmission spectra is very important to evaluate the performance of the proposed sensor at different concentrations of H_2_SO_4_. Figure 5 illustrates the behavior of the ternary local resonant PSi-PnC with varying concentrations of H_2_SO_4_. All modes are moved to the left towards the low frequencies in the same trends as the concentration of H_2_SO_4_ increases from 0 to 15%. The resonance frequency decreases as the concentration of H_2_SO_4_ increases. As seen in Figure 5, the resonance mode decreases from 3.6548 × 10^8^ Hz to 3.6160 × 10^8^ Hz as the H_2_SO_4_ concentration increases from 0% (pure water) to 16%.

The behavior of the amplitude at different concentrations does not follow the same trend. This behavior can be understood using the data presented in Figure 2 and Equations (15) and (16). From Figure 2, the density of the solution increases semi-linearly with a rate of approximately Δρ/Δc = 6.7 as the concentration of H_2_SO_4_ increases. On the other hand, the speed of sound exhibits a quartic polynomial relationship with the concentration as expressed by Equation (16). It is important to note that the rate of increase of sound speed with respect to concentration (Δv/ΔC) is not uniform across the concentration range of 0 to 15%, as depicted in Figure 2. The values of sound speed and density have an effect on the amplitude of the resonance mode. Hence, the behavior of acoustic wave amplitude for resonance at different concentrations does not take the same trend. In other words, as the concentration of the H_2_SO_4_ changes, both the density and speed of sound change in a non-uniform manner. This shows that the relationship between concentration and amplitude acoustic wave behavior is complex and cannot be simply modeled by a linear or polynomial function.

There are several parameters considered, including sensitivity (S), the figure of merit (FoM), quality factor (QF), damping rate (γ), full width at half maximum (FWHM), and detection limit (DL), which are used to study the performance of a sensor. The LR peak’s position (f_r_) and width (FWHM) at each H_2_SO_4_ concentration strongly influence the performance parameters [20]. This section calculates and discusses these performance parameters, which can be defined using the following relationships [63,64].

(18) $$FoM=\frac{S}{FWHM}$$

(19) $$QF=\frac{f_{r}}{FWHM}$$

(20) $$DL=\frac{f_{r}}{20\;S\;QF}=\frac{0.05}{FoM}$$

(21) $$\gamma =\frac{FWHM}{2\;f_{r}}=\frac{0.5}{QF}$$

The FWHM is related to the sensitivity and selectivity of the sensor. A narrower FWHM indicates a higher resolution and better selectivity. This means that the sensor can distinguish between different frequencies of waves with greater accuracy, allowing it to detect small changes in the stimulus. The QF in phononic sensors is a measure of the energy stored in the sensor compared to the energy lost due to mechanical dissipations in the system. A higher QF indicates that the sensor has high sensitivity and selectivity [65]. In other words, the QF measures the sensor’s selectivity level in detecting a specific stimulus. The QF is determined by the sharpness of the resonant peak in the transmission spectrum of the sensor. The QF is inversely proportional to FWHM, which means that a narrower FWHM leads to a higher QF. The FoM is used to evaluate the overall performance of a sensor. A higher FoM indicates a more effective sensor with better overall performance, including higher sensitivity and selectivity [66]. The DL refers to the smallest gas concentration a sensor can detect with a certain level of accuracy and precision [67]. In other words, the DL is the lowest gas concentration that a sensor can reliably distinguish from zero. The damping rate (γ) measures the rate at which the sensor’s resonant frequency decreases over time due to energy losses. A lower damping rate leads to higher sensitivity, as it allows the sensor to detect even small changes in the stimulus.

**Figure 6:**
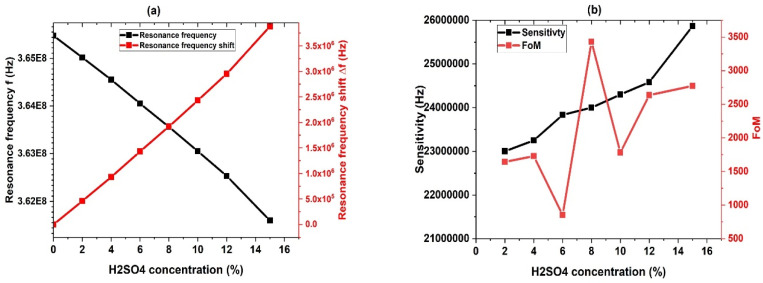

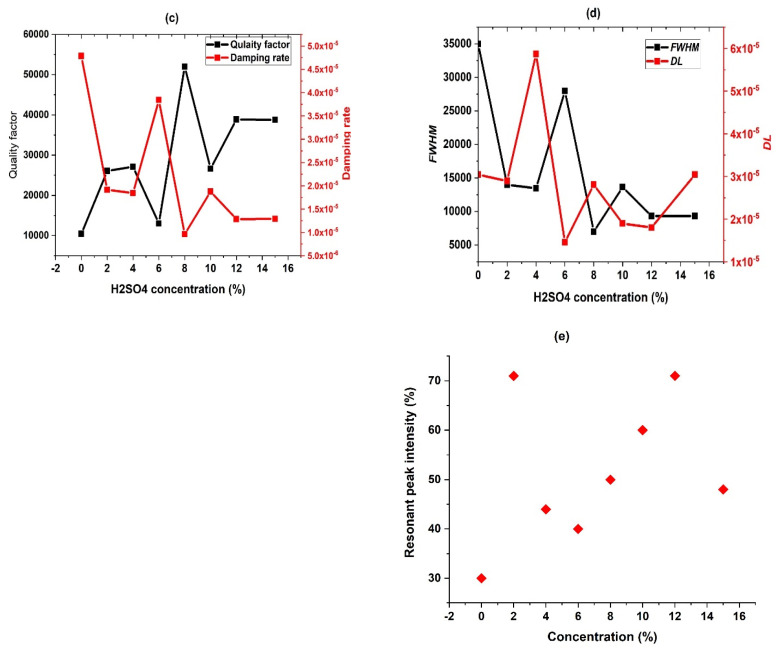
The effect of different concentrations of H_2_SO_4_ on the sensor performance parameters (**a**) Resonant peak position and resonant frequency shift ∆f, (**b**) Sensitivity and FoM, (**c**) QF and damping rate, (**d**) FWHM and DL and (**e**) Resonant peak intensity.

The performance of the phononic sensor is graphically depicted in Figure 6 based on the numerical results of Equations (18)–(21). The resonance frequency decreases as the concentration of H_2_SO_4_ increases, as indicated in Figure 6a. The resonance mode shifts $\left(\left|∆f=f_{r}-f_{w}\right|\right)$, which increases from 0 to 3.88 × 10^6^ Hz as the H_2_SO_4_ concentration changes from 0% (pure water) to 15%. As the concentration of H_2_SO_4_ increases, the sensitivity increases, as shown in Figure 6b. This trend is captured by a polynomial fitting of the numerical results, as shown in Figure 3. The maximum sensitivity, about 2.5867 × 10^7^ Hz, is attained at an H_2_SO_4_ concentration of 15%. Figure 6b displays the dependence of the FoM on the concentration of the H2SO4 solution, with the highest FoM value of 3.431 × 10^7^ being achieved at an H_2_SO_4_ concentration of 8%. This suggested the best performance of the sensor at this concentration.

Figure 6c displays the variation of QF with changes in H_2_SO_4_ concentration. The QF value depends on the spectral position of LR and the full width at half maximum (FWHM). The highest QF value of 51,974 is achieved at an H_2_SO_4_ concentration of 8%, where the smallest FWHM value is also observed. A high QF indicates minimal losses or damping at the resonance frequency, which is crucial for designing a precise and accurate sensor. Based on Equation (21), the damping is inversely proportional to QF. Damping can negatively affect the structure’s performance by dissipating energy. Figure 6c shows that the damping rate values range between 0.4785 × 10^−4^ and 0.0962 × 10^−4^. This finding suggests that the damping rate is very small and can be neglected, which is beneficial for the sensor’s response to the input signal [68,69]. 

Typically, a narrow peak has a high-quality factor (QF) and a low damping coefficient. As shown in Figure 6d, the high and low values of the FWHM are 3.4976 × 10^3^ and 0.9326 × 10^3^ Hz, corresponding to 15% and 0% H_2_SO_4_ concentration, respectively. The Figure 6d also demonstrates that the DL is very small. The DL values less than 0.3042 × 10^−4^ indicate the sensor’s efficient performance.

The FWHM does not exhibit a uniform change with increasing H_2_SO_4_ concentration. The anomalous behavior observed in other parameters such as FOM, QF, damping rate, and DL can be attributed to irregular changes in FWHM based on Equations (18)–(21). Despite the complex and irregular behavior of these parameters, the calculated values for them are considered good and indicate that the efficiency of the sensor is excellent. This suggests that our sensor can effectively detect changes in H_2_SO_4_ concentration, even in the presence of fluctuations in FWHM and other related parameters.

Concerning the analysis of the transmitted signals as a function of the amplitude of acoustic waves under different concentrations is plotted in Figure 6e. As shown in this figure, the amplitude of the transmitted signals (transmission intensity of the resonant peak) versus H_2_SO_4_ concentrations follows irregular behavior. The transmission intensity of concentrations 0, 2, 4, 6, 8, 10, 12, and 15% are 30, 71, 44, 40, 50, 60, 71, and 48%, respectively. The most important thing is that all peaks have high acoustic transmission intensity and then high acoustic energy confinement through the pores of the Psi layers. The irregular transmission intensities are related to changes of the acoustic path difference of each peak [52,55]. This path difference is determined by the well-known Bragg’s diffraction rule;

(22) $$2d\sin\theta =n\frac{c}{f}$$

where, d is the layer thickness, n is an integer, c is the sound speed, and *f* is the frequency. Based on this rule, the condition of constant phase difference must be applied for each concentration value. Therefore, the speed of sound in each Psi layer is directly connected to the frequency value of the resonant peak, where, at each specific concentration, the density, and sound speed change, as well, which, in turn, alter the acoustic wave path length, frequency position, and intensity of the resonant peak.

### 3.6. Effect of Temperature on the Performance of the Phononic Sensor

As reported in much experimental research, the temperature affects the acoustic properties, especially the sound speed of H_2_SO_4_, only at high temperatures and concentrations. At C = 10% of H_2_SO_4_, the depend on acoustic speed and density of H_2_SO_4_ aqueous solution to change temperature from 50 °C to 130 °C can be described using Equations (23) and (24) [70,71,72,73].

(23) $$v\left(m/s\right)=1600.5-0.45\;T\;({}^{\circ}C)$$

(24) $$\rho \;(Kg/m^{3})=1099.5-0.65\;T\;({}^{\circ}C)$$

**Figure 7:**
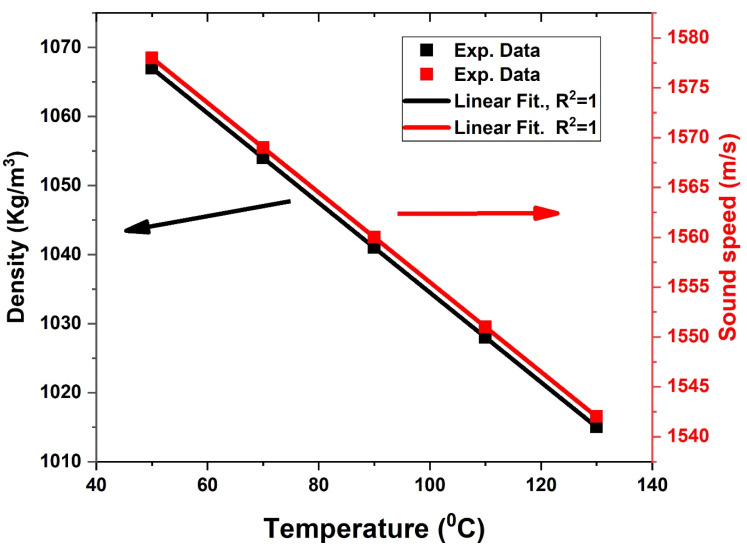
Acoustic properties (density and sound speed) of the sulfuric acid aqueous solution versus temperatures at a concentration of 10%.

**Figure 8:**
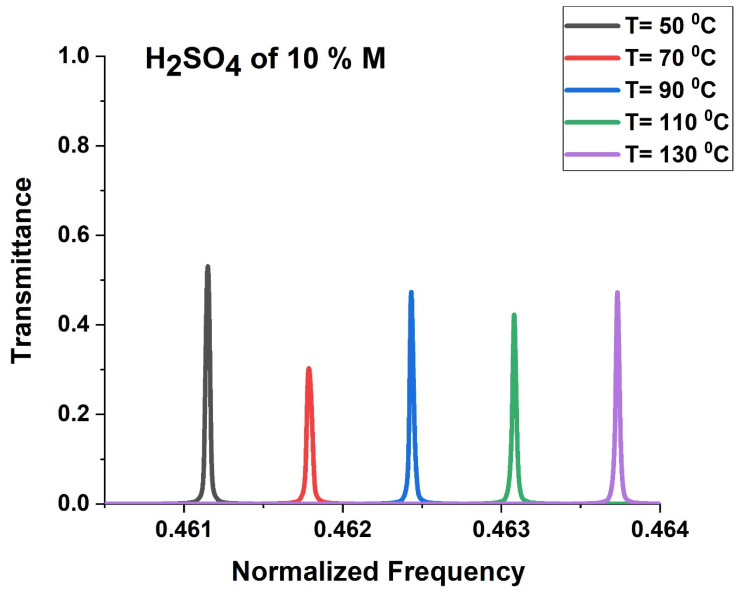
The transmission spectra of the ternary local resonant PSi-PnC at different temperatures of H_2_SO_4_.

Figure 7 clarifies that the density and acoustic wave velocity of the aqueous solution of H_2_SO_4_ decrease linearly with the increased temperature. This Figure was plotted at a constant concentration of 10%. Here, the density decreases from 1066 kg/m^3^ at 50 °C to 1037 kg/m^3^ at 95 °C. The acoustic speed of H_2_SO_4_ is 1569 m/s m/s at 70 °C, which is higher than that at 110 °C (1550 m/s). Figure 8 displayed the transmission spectra of the ternary LR PSi-PnC with different temperatures (50–130 °C) of H_2_SO_4_ at C = 10%. As shown in Figure 8, the position of the resonant mode is shifted towards a higher frequency due to the decrease in the acoustic properties of H_2_SO_4_ with increasing temperature.

### 3.7. Effect of Temperature on Performance

**Figure 9:**
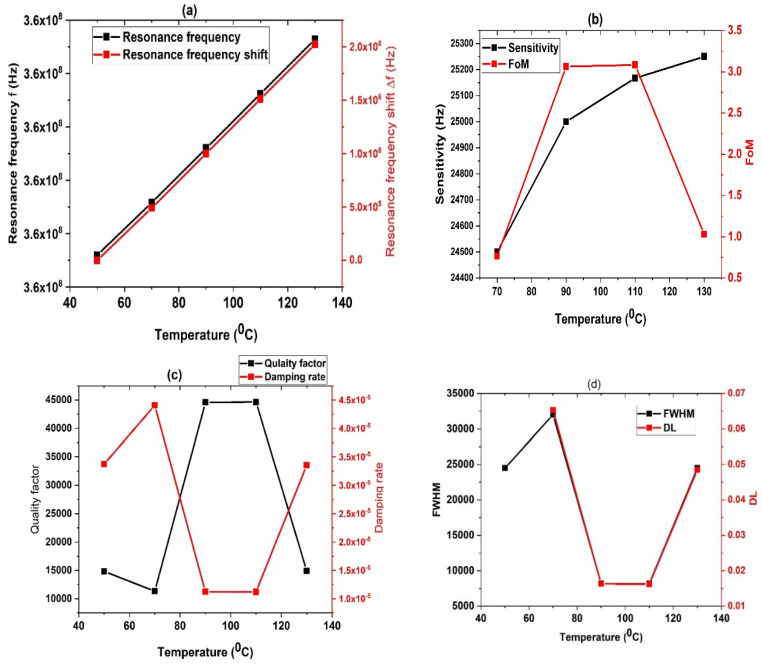
The effect of temperature on the performance parameters of the H_2_SO_4_ Psi PnC sensor. (**a**) Resonance frequency and frequency shift, (**b**) sensitivity and FoM, (**c**) quality factor and damping rate, and (**d**) FWHM and DL.

Figure 9 illustrates the impact of temperature on the performance parameters of the 1D ternary PSi-PnC sensor at different temperatures. The data was collected by comparing various temperature ranges against the reference temperature of 50 °C. As illustrated in Figure 9, both the resonance frequency and sensitivity increase with temperature. This phenomenon can be explained by the decrease in density and speed of sound with increasing concentrations of H_2_SO_4_. However, the behavior of the parameters FOM, QF, damping rate, and DL is irregular and non-uniform, which can be attributed to the anomalous changes in FWHM at different H_2_SO_4_ concentrations, as described in our previous findings. Figure 9a shows that as the temperature increases from 50 °C to 130 °C, the peak position of the sensor moves from f = 3.628 × 10^8^ Hz to f = 3.6482 × 10^8^ Hz. Additionally, the resonance frequency shift exhibits a linear increase with temperature. The sensitivity increases from 2.45 × 10^4^ Hz/°C to 2.525 × 10^4^ Hz/°C as the temperature rises from 50 to 130 °C as illustrated in Figure 9b. The FoM ranges from 0.7652 to 3.0838 with temperature changes.

In Figure 9c, the quality factor (QF) and damping coefficient (γ) are presented as a function of temperature, with the two parameters exhibiting opposite behavior. The QF remains higher than 1.4900 × 10^4^, while the γ remains lower than 0.4407 × 10^−4^. The FWHM and DL are shown in Figure 9d. Overall, these results suggest that our proposed sensor is a promising platform for H_2_SO_4_ detection and sensing.

Table 2 presents a comprehensive comparison between the sensitivity values obtained from our proposed phononic sensor and those reported in previous studies. Our results demonstrate that our phononic sensor exhibits a significantly higher sensitivity compared to numerous previously published sensors [72,73,74,75,76,77,78,79,80]. Moreover, our phononic sensor has several distinct advantages over other sensors in the market. Firstly, it is fabricated using readily available and cost-effective materials, making it an affordable option for a wide range of applications. Additionally, the fabrication process is relatively simple and does not require any complex or specialized equipment, resulting in a more straightforward and efficient manufacturing process. The sensor’s high sensitivity is attributed to the unique properties of the phononic crystal structure, which enables the efficient manipulation of acoustic waves and the detection of subtle changes in the surrounding environment. This suggests that the proposed phononic sensor holds great promise for a wide range of sensing applications, making it a promising new sensor platform.

The optimization techniques and tools are very important to enhance the performance of complex systems and determine their optimal efficiency. They can be used for the optimization of proposed biosensor structures in our future research to obtain the best performance. In this regard, statistical optimization techniques such as the design of experiments (DOE), response surface methodology (RSM), and Taguchi methods employ statistical analysis to optimize a system or process [79]. Numerical optimization algorithms like Newton’s method, conjugate gradient method, genetic algorithm, and Nelder-Mead method can also be used to optimize biosensors based on numerical modeling [80]. Additionally, artificial intelligence (AI) and machine learning (ML) are recently increasingly for optimizing the performance of sensor systems [81]. By leveraging these advanced techniques, sensors can be optimized to achieve the desired performance levels and meet the demands of various applications.

## 4. Conclusions

In this paper, we presented a tunable porous silicon one-dimensional PnC structure as a liquid sensor. The sensor configuration is arranged as {silicone rubber /[PSi_1_/PSi_2_/PSi_3_]^N^/silicone rubber}. The studied sensor is presented for detecting a very important chemical acid represented by sulfuric acid (H_2_SO_4_) in a rare-used adopted range of concentrations (0–15%). In general, silicon can withstand the presence of H_2_So_4_ rather than other mechanical materials used in PnCs, such as epoxy and nylon, as sulfuric acid can affect these materials over the time, even with low concentrations. Additionally, silicon has a very high elastic constant to withstand sulfuric acid easily. On the other hand, silicone rubber is a highly chemically stable and low-cost material (with high melting temperature), and meanwhile is one of the preferred polymers that can generate local resonant modes easily. These modes are the main compass through the detection process. The modes shifted to new frequency positions by changing the sulfuric acid concentrations. Furthermore, we studied the optimum PnC sensor, including binary Bragg band gaps PnCs, ternary Bragg band gaps PnCs, binary local resonant PnCs, and ternary local resonant PnCs. In this regard, the sensor has provided a super sensitivity compared with many PnCs sensors with the value of 2.3 × $10^{7}$ Hz for a concertation change of just 2%.

Further, the other performance parameters are superb, such as a quality factor of 2.6 × $10^{4}$, figure of merit of 1.6 × $10^{3}$, very low detection limit of 0.28 × $10^{-4}$, and very low damping rate of 0.1916 × $10^{-4}$. Even though some previous defective 1D PnCs achieved similar sensitivity, they depend on a very high range of concentration up to 100%. Additionally, they have provided very high FWHM. Additionally, the temperature effects have been taken into consideration. These advantageous factors should be considered in any future investigations and experimental studies of the optimum fluidic sensors based on phononic crystals.

## Figures and Tables

**Table 1:** Densities, acoustic sound speeds, and thicknesses of the proposed sensor’s materials in the absence of the aqueous H_2_SO_4_ solution [45,46,47].

Material	Density (kg/m^3^)	Acoustic Sound Speed (m/s)	Thickness (μm)
**PSi_1_ (P = 20%)**	2063.6	7741.1 [45,46]	6
**PSi_2_ (P = 40%)**	1797.4	7088.3 [45,46]	3
**PSi_3_ (P = 60%)**	1530.9	6210.1 [45,46]	1.5
**Silicon Rubber**	1.30	25 [46]	0.01–0.20
**Water**	1001	1507 [47]	—
**H_2_SO_4_ salt**	1830	1253 [47]	—

**Table 2:** A comparison between our proposed sensor sensitivity with previous PnC sensors.

Structure	Liquid/Gas	Sensitivity	Ref
2D-PnC with ring resonator cells	Methanol	12.54 Hz/%	[72]
Multilayer periodic structure 1with a defect layer	Organic compounds	0.1614 × 10^7^ MHz/(ms^−1^)	[73]
Stainless steel plate with cylindrical holes and a resonant cavity	Ethanol	0.91 × 10^3^ Hz/(ms^−1^)	[74]
2D periodic arrangement of cylindrical holes	Propanol	0.4 × 10^3^ Hz/(m s^−1^)	[75]
Triangular lattice PnC	Temperature	4.4 × 10^3^ Hz/K	[76]
2D-PnC crystal slab with defect	Propanol	0.39 × 10^7^ Hz/m s^−1^	[77]
1D-PnCs with defect layer	Glycine	969.9 × 10^3^ Hz	[78]
Ternary locally resonant porous silicon 1D-PnC	Sulfuric acid	2.5867 × 10^7^ Hz	This work

## Data Availability

Requests should be addressed to any author.
